# Effect of Light- and Dark-Germination on the Phenolic Biosynthesis, Phytochemical Profiles, and Antioxidant Activities in Sweet Corn (*Zea mays* L.) Sprouts

**DOI:** 10.3390/ijms18061246

**Published:** 2017-06-10

**Authors:** Nan Xiang, Xinbo Guo, Fengyuan Liu, Quan Li, Jianguang Hu, Charles Stephen Brennan

**Affiliations:** 1 School of Food Science and Engineering, South China University of Technology, Guangzhou 510641, China; fexnan@mail.scut.edu.cn (N.X.); fernyarn@gmail.com (F.L.); quanliai01@163.com (Q.L.); 2 Crop Research Institute, Guangdong Academy of Agricultural Sciences, Guangzhou 510640, China; jghu2003@263.net; 3 Key Laboratory of Crops Genetics Improvement of Guangdong Province, Guangzhou 510640, China; 4 Department of Wine, Food and Molecular Bioscience, Lincoln University, Canterbury 7647, New Zealand

**Keywords:** germination, sweet corn, phenolic biosynthesis, antioxidant activity

## Abstract

Sweet corn is one of the most widely planted crops in China. Sprouting of grains is a new processes to increase the nutritional value of grain products. The present study explores the effects of light on the nutritional quality of sweet corn sprouts. Gene expression of phenolic biosynthesis, phytochemical profiles and antioxidant activity were studied. Two treatments (light and dark) were selected and the morphological structure of sweet corn sprouts, as well as their biochemical composition were investigated to determine the effects of light on the regulation of genes responsible for nutritional compounds. Transcription analyses for three key-encoding genes in the biosynthesis of the precursors of phenolic were studied. Results revealed a negative regulation in the expression of *_Zm_PAL* with total phenolic content (TPC) in the light group. TPC and total flavonoid content (TFC) increased during germination and this was correlated with an increase in antioxidant activity (*r* = 0.95 and 1.0). The findings illustrate that the nutritional value of sweet corn for the consumer can be improved through germination to the euphylla stage.

## 1. Introduction

Epidemiological experiments have demonstrated a relationship between the consumption of food with high quantities of phenolic compounds and a reduction in the risks of chronic and degenerative diseases, such as cancers and cardiovascular disease [1,2,3]. Phenolic compounds and flavonoids are natural antioxidants, anti-aging agents, and are able to reduce inflammation [4,5]. The antioxidant activities of phenolics and flavonoids have been shown to be a result of their regulation on PGE2 synthesis and inhibition on the expression of *NF-κB* gene [6]. It is accepted that fruits and vegetables are rich in bioactive nutrients; for instance, sweet corn contains a number of phytochemicals, as well as dietary fiber, protein, and carbohydrates [7]. Plant-based foods have been shown to provide benefits in preventing consumers from chronic diseases [2]. The consumption of a variety of fruits and vegetables are recommended in the daily diet by the 2015–2020 dietary guidelines for Americans [8] to obtain a plethora of plant-based phytochemicals. 

De novo synthesis of phytochemicals often occurs during germination of plant seeds and plays an important role in the prevention of chronic diseases. For instance, broccoli sprouts have been investigated extensively and reported to have a positive anti-cancer effect [9]. These phytochemicals can be increased during germination, as has been shown by Guo et al. [6] who observed a 4.5-fold increase in total phenolic content during an eight-day germination of mung bean sprouts. Similarly Gan et al. [10] reported increases in phenolic contents. The potential health-promoting benefits of sprouted grains have received much attention [11]. Recently many researchers have investigated how germination can manipulate the chemical composition of seeds and enhance the amount of bioactive ingredients [12] with research being conducted on germinating brown rice [13], buckwheat [14], soybean [15], and onion [16]. Previous research has shown that germination of seven days can increase total phenolic content (TPC) and total flavonoid content (TFC) levels in buckwheat, illustrating a linear relationship between phenylalanine ammonia-lyase (PAL) activity and flavonoid accumulation [14]. However, some seeds show contrasting behaviour; for instance, germination may decrease the levels of TPC and TFC in kidney beans [17]. This illustrates that the mechanisms of phytochemical development during germination varies between plant species.

Researchers have characterized the genes and regulation of phenolic compound biosynthesis [18]. For instance, PAL has been shown to mediate the first step of the phenylpropanoid pathway and catalyse the transformation from phenylalanine to cinnamic acid [19]. Chlorogenic acid is subsequently generated without any enzymes on the basis of cinnamic acid. Subsequently, cinnamic acid is transformed to *p*-coumaric acid through the action of action of *C4H* gene-encoded enzyme cinnamate-4-mono-oxygenase (C4H). Similarly, 4-coumaroyl CoA is catalysed by 4-coumaroyl:CoA-ligase (4CL) which, in turn, is encoded by the *4CL* gene. It follows, therefore, that *PAL* can be regarded as a key gene to the phenolic synthesis pathway [20]. Thus, the investigation on the expression of the three genes can unambiguously characterize the phenolic profiles during seeds germination.

Sweet corn is an abundant vegetable in China and consumed daily for its nutritional value, including phytochemicals and antioxidant properties [7]. It is widely planted in East China [21]. There is a plethora of food products derived from sweet corn in China, including dried kernel, fresh cobs, and sweetcorn juice, thus, our research focused on the potential to utilize plant biosynthetic pathways in developing a novel nutritious food product. Germinated sweet corn sprouts have been studied recently as a novel method of biotechnological processing to improve the kernel eating quality, however, the effect of germination on phytochemicals of sweet corn seeds has not been studied extensively. A previous study reported that the TPC and antioxidant activity increased with germination of sweet corn sprouts [22]. This study characterized the regulation of related enzymes responsible for phenolic production, but did not explain the relationship between the enzymes and relevant gene expression. Our study aims to systematically study the profiles of phenolic biosynthesis and antioxidant activity values of sweet corn seeds during germination, and characterizes the mechanism of the raised nutritional value in order to raise a novel edible method of sweet corn. The germination of sweet corn seed was morphologically divided into four stages: radicle stage, germinal stage, fibril stage, and euphylla stage. Germination was also conducted in both light and dark treatments to investigate macroscopic differences during such germination and how this affected gene expression and antioxidant activity.

## 2. Results

### 2.1. Effect of Germination on Moisture Content of Samples

The moisture contents of each stage of sample under light treatment were 53.51%, 63.31%, 63.80%, and 74.33% at radicle, germinal, fibril, and euphylla stages, respectively. In contrast, the moisture content of the dark samples was lower, with 50.42%, 58.41%, 65.64% and 70.27% at each stage.

### 2.2. Effect of Germination on Relative Gene Expression Profiles of Phenolic Biosynthesis

**Figure 1:**
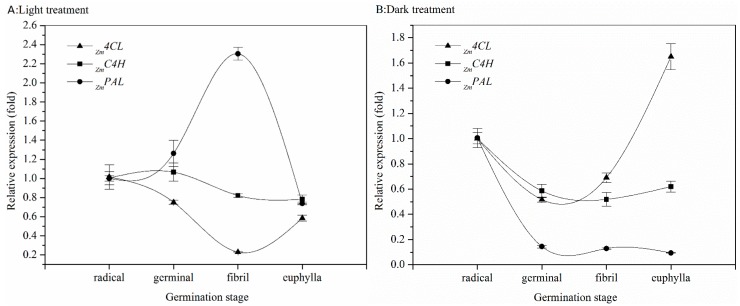
Effect of germination on expression of genes involved in phenolic biosynthesis (mean ± SE, *n* = 3): (**A**) light treatment; and (**B**) dark treatment. *_Zm_4CL*: *4-coumaroyl:CoA-ligase*; *_Zm_C4H*: *cinnamate-4-hydroxylase*; *_Zm_PAL*: *phenylalanine ammonia lyase*.

The related genes encoding for enzymes involved in the metabolites biosynthesis were analyzed by real-time PCR and results are listed in Figure 1A (light group) and Figure 1B (dark group). The kernels germinating under light stimulation showed the greatest *_Zm_PAL* expression at the fibril stage. However, a significant decline happened after the peak, with *_Zm_C4H* expressed less in later periods and the greatest expression can be considered at the germinal stage. The lowest transcript expression of *_Zm_4CL* was observed at the fibril stage of germination (Figure 1A). Conversely, the highest level of *_Zm_PAL* expression was observed at this stage. Without the stimulation of light, the expression of *_Zm_4CL* was not stimulated until the fibril stage and the greatest expression was at the euphylla stage. The two other genes were detected at lower expression quantities in the sequence period.

### 2.3. Effect of Germination on Total Phenolic Content (TPC) in Light and Dark Treatments

**Figure 2:**
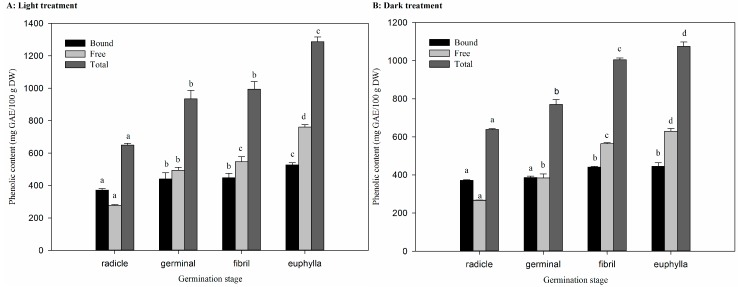
Phenolic content of sweet corn during germination under light and dark treatment (mean ± SD, *n* = 3): (**A**) light treatment; and (**B**) dark treatment. Bars with no letters in common in each fraction are significantly different (*p* < 0.05).

The changes of total phenolic contents of sprouts under light treatment can be observed in Figure 2A. Free phenolic contents were significantly increased (*p* < 0.05) in a time-dependent manner during germination and reached up to 759.8 ± 15.9 mg gallic acid equivalent (GAE)/100 g dry weight (DW) at the euphylla stage, which was approximately 2.5 times higher than the value observed at the radicle stage. The contribution of the free form was increased from 42.74% to 59.02%. The increase of bound phenolic in sweet corn during germination also occurred, except in the final stage of germination. Therefore, the total phenolic contents increased with passing time, and were doubled (1287 ± 28 mg GAE/100 g DW) at the euphylla stage.

The effect of germination on total phenolic content in dark-germinated sweet corn sprouts is shown in Figure 2B. The free phenolic contents contributed from 41.81% to 58.54%, similar to the observation of the light group, as the value rose to 629.0 ± 14.0 mg GAE/100 g DW at the euphylla stage. The bound phenolic compounds in sweet corn sprouts showed a steady increase during germination. With the increase in both free and bound phenolic content, the value of total phenolic content rose to 1075 ± 23 mg GAE/100 g DW in the last two germination stages compared to the radicle stage.

### 2.4. Effect of Germination on TFC in Light and Dark Treatments

**Figure 3:**
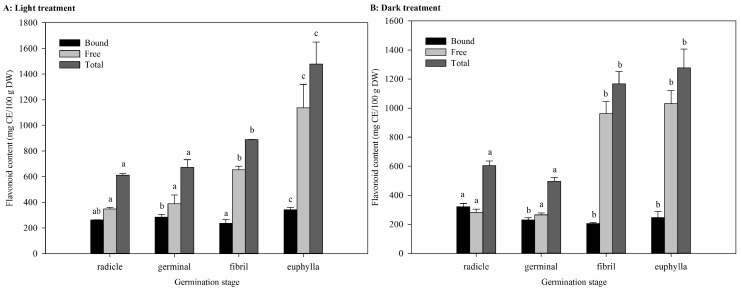
Flavonoid content of sweet corn during germination under light and dark treatment (mean ± SD, *n* = 3): (**A**) light treatment; and (**B**) dark treatment. Bars with no letters in common in each fraction are significantly different (*p* < 0.05).

The changes of flavonoid values of sweet corn during germination are shown in Figure 3 under light and dark treatment, respectively. In the light group, a three-fold increase of the free flavonoid content at the euphylla stage was observed compared to the radicle stage. On the contrary, significant changes were not detected in the bound flavonoids. The value of total flavonoids doubled from 611.6 ± 12.6 mg catechin equivalent (CE)/100 g DW at the radicle stage to 1479 ± 170.6 mg CE/100 g DW at the euphylla stage. The free flavonoids increased to 80.70% of total at the latter stages of germination under dark treatment. Compared to the radicle stage, the free flavonoid content at the euphylla stage reached a peak and constant value of 1031 ± 90 mg CE/100 g DW which is double that of the radicle stage. A decrease was detected in the bound flavonoid content and its level was reducing to 19.30% of the total. Total flavonoids value which is 1277 ± 129 mg CE/100 g DW at the euphylla stage, acting two times higher than the value at early stages.

### 2.5. Effect of Germination on Phenolic Profiles in Light and Dark Treatments

Five phenolic compounds, including gallic acid, chlorogenic acid, syringic acid, hydroxycinnamic acid, and ferulic acid, were identified in both free and bound forms in sweet corn sprouts, as presented in Table 1. HPLC analyses indicated changes did not occur in the composition during germination, but that the content changed at different stages (*p* < 0.05). For instance, the content of gallic acid in the free form had no significant changes during germination in the two conditions, while an apparent increase can be detected in the bound form (*p* < 0.05). The value of the bound gallic acid content at the euphylla stage (74.40 ± 3.04 mg/100 g DW) was observed at the radicle stage under light treatment (63.88 ± 6.02 mg/100 g DW). The level of the bound form increased from 23.92% to 43.88% as a percentage of the total content. Therefore, the trend of total content of gallic acid during germination is similar to the bound form. Chlorogenic acid was not detected in the bound extracts, existing only in the free form, and its content increased during growing stages as 40.72 ± 1.01 mg/100 g DW in the light exposed group, and 29.28 ± 0.42 mg/100 g DW in the dark group at the last stage. Interestingly, the two groups had similar contents of chlorogenic acid at first, however, germination under different conditions caused a larger accumulation of content in the group exposed to light than in the dark group. Syringic acid in the bound form was not detected during the early stages of sprouting. The total content of syringic acid increased at the germinal stage was comparable to the radicle stage, and increased at the latter stages of germination with the highest value expressed at the euphylla stage as 28.36 ± 0.08 mg/100 g DW (light) and 22.14 ± 0.76 mg/100 g DW (dark). Accumulations of hydroxycinnamic acid occurred in both free and bound forms. Under the light treatment, the value rose from 74.42 ± 2.87 to 134.1 ± 2.6 mg/100 g DW in total. Sprouts in dark group rose to a value of 122.6 ± 2.7 mg/100 g DW at the euphylla stage, which was double the value observed at the radicle stage. The free form of ferulic acid also showed a significant accumulation with the increase in the germinating time, from 14.48 ± 0.37 mg/100 g DW at radicle stage to 27.30 ± 0.36 mg/100 g DW at the euphylla stage under light treatment, and from 13.99 ± 0.62 to 22.12 ± 0.29 mg/100 g DW under dark treatment.

### 2.6. Effect of Germination on Antioxidant Activity in Light and Dark Treatments

**Figure 4:**
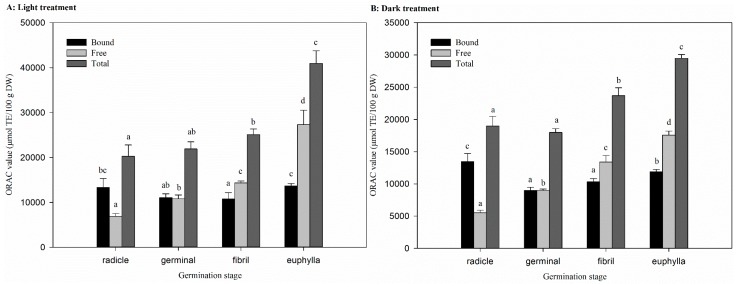
Antioxidant activity of sweet corn during germination under light and dark treatment (mean ± SD, *n* = 3): (**A**) light treatment; and (**B**) dark treatment. Bars with no letters in common in each fraction are significantly different (*p* < 0.05).

The antioxidant activity of sweet corn during the germination period under light is illustrated in Figure 4. In free-form extracts, the antioxidant activity increased with time and attained 27,305 ± 3220 μmol trolox equivalent (TE)/100 g DW at the euphylla stage, which represented a four-fold increase compared to the value at the radicle stage (6893 ± 624 μmol TE/100 g DW). Antioxidant activity in the bound form occupied 65.96% of the total at the radicle stage but reduced with time to 33.33% at the euphylla stage. In total, the antioxidant activity was observed to peak at the euphylla stage (40,956 ± 2822 μmol TE/100 g DW), which was double that of the first stage. The changes of antioxidant activity in the dark group were similar to the light group, as shown in Figure 4. The free form extractions had the best antioxidant activity at the euphylla stage (17,579 ± 628 μmol TE/100 g DW), which was five times higher than at the radicle stage (5518 ± 410 μmol TE/100 g DW). A decreased contribution occurred from radicle to euphylla stages of antioxidant activity in the bound form, which was reduced from 70.94% to 40.35% of the total. In all, the total antioxidant activity was significantly enhanced to a value as 29,471 ± 590 μmol TE/100 g DW (*p* < 0.05).

## 3. Discussion

Phenolic compounds are an essential metabolite family in plants, and play important roles in a plant’s life [6]. The results showed that TPC increased in a time-dependent manner in both light and dark treatment groups in sweet corn sprouts during germination. The results illustrated a correlation (*r* = 1.0) between free form phenolic compounds and TPC in both groups, which indicated the accumulation of TPC was owing to the increase of phenolics in free extracts. A relationship (*r* = 0.96) between TPC and TFC was also observed, which concluded that an increase of TPC was mainly from the increase of TFC. Previously, it had been reported that TPC increased during germination by about 7.4-fold as compared to raw seeds [23]. Other similar effects of germination on phenolic content have been observed in brown rice [13] and buckwheat [14], which indicate a higher nutritional value of germinated seeds. Although the accumulation occurred in both treatments, the sweet corn sprouts tend to have a lower content of phenolics in the latter stages of germination in the dark samples compared to light. 

Flavonoids are a major component of phenolic compounds and are useful in protection from chronic diseases, such as cardiovascular disease, cancer, and diabetes [6]. As well as playing a role in pigmentation of flowers, fruits, and seeds, flavonoids play important roles in signaling, transport, and protecting against microbes and UV [24]. Our results exhibit that the free form of flavonoids comprise the major component of the total flavonoids. The accumulation of TFC was time-dependent; similar to observations previously recorded in mung bean, radish, broccoli, and sunflower [11]. The flavonoid content in free form was positively correlated with TFC (*r* = 1.0) in the two treatments. A negative correlation between flavonoid content in free and bound extracts was detected, which depicted a transformation of flavonoids from free to bound forms.

Enhanced antioxidant activity in processed sweet corn was first demonstrated by Dewanto et al. [25]. Other reports have illustrated an increase of antioxidant activity during germination in rice bean sprouts [26] and mung bean [6]. Our study also detected an enhancement in sweet corn sprouts, which indicated another processing method on raising the nutritional value of the consumption of sweet corn. The relationship between the increase of phenolic content and the enhancement of antioxidant ability during germination has been demonstrated previously in canary seed (*Phalaris canariensis* L.) [23]. The Pearson’s correlation analysis of our results showed a high relationship between TFC and antioxidant activity in both groups in our study (*r* = 0.95 and 0.94), respectively.

Gene expression can explain the mechanism of the variation of phenolics. PAL plays an important role in the first transformation of phenolic biosynthesis pathway, catalyzing phenylalanine to cinnamic acid [27]. The following step required the action of C4H to generate p-coumaric acid, a precursor of phenolic or flavonoid. Therefore, 4CL is a vital enzyme activating on the biosynthesis of flavonoid. According to the results shown in Figure 1, the changes of the content of phenolic compounds during germination of sweet corn sprouts and seedlings can be explained as follows: At the beginning, *_Zm_PAL*, *_Zm_C4H*, and *_Zm_4CL* successively catalyze the conversion of phenylalanine to *p*-coumaroyl CoA and results in an accumulation of total phenolic. With the increase in the expression of *_Zm_PAL*, the contents of phenolic compounds were higher than that at the beginning of cultivation. For example, the gallic acid in bound form of extracts showed a doubled accumulation in the euphylla stage compared to the radicle stage. Similar increases were also observed in total chlorogenic acid, syringic acid, and hydroxycinnamic acid. The free form of ferulic acid had a two-fold increase. The increases of these phenolic compounds resulted in inhibitions in following period of the expression of genes encoding for involved enzymes. A negative regulation in hormonal regulation in biology was observed, and that phenolic compounds are regulators of gene expression illustrated previously can support the phenomenon in our study [28]. Therefore, when sprouts grew to seedlings at the euphylla stage, the expression of the first enzyme which activated the biosynthesis reaction dramatically reduced. On the contrary, the enzymes that catalyzed the following synthesis mechanism were better expressed because of the accumulation of a precursor of phenolic compounds: cinnamic acid. As a result, TFC were aggrandized in latter stages. In the whole germination period of sweet corn seeds, TPC increased in a time-dependent manner, but at different rates, due to the variation in the expression of genes. A previous study demonstrated an increase in the expression of *PAL* during the initial germination period could promote the accumulation of phenolic acids in flaxseed [29]. Without the stimulation of light, significant changes of TPC did not occur from the radicle to germinal stages, which was associated with a lower expression of relative genes. Only when fibrils were produced was the reaction of creating phenolic observed to be rapidly stimulated, which is a result of the added expressional quantity of the three related genes. *_Zm_PAL* and *_Zm_C4H* expressed a maximum quantity at the radicle stage and declined in subsequent periods. The expression of *_Zm_4CL* increased during germination and achieved a maximum value at the euphylla stage. At the beginning of germination, the expressions of genes were merely the same in both groups. However, after cultivating in the dark for a period, a low expressed activity of genes was detected and showed a slow increase of total phenolic. Numerous studies have researched the influences of light during germination of plants. Researchers have concluded an enhancement in TPC with an illumination of blue LED light in pea sprouts [30] and an accumulation of phenolic compounds in Tartary buckwheat sprouts under LED light has been detected beyond gene expression [31]. Previously it was shown that a correlation (*r* = 1.0) between TPC and oxygen radical absorbance capacity (ORAC) values was recorded in canary seed [23], which is similar to our observations.

## 4. Materials and Methods

### 4.1. Chemicals

Ascorbic acid (ASA), catechin, chloranil, 2,6-dichloroindophenol sodium salt hydrate (DIP), 2,2′-azobis-amidinopropane (ABAP), Trolox, Folin-Ciocalteu reagent, and gallic acid (GA) were purchased from Sigma (St. Louis, MO, USA). Metaphosphoric acid, acetone, alcohol, hydrochloric acid (HCl), ethyl acetate, sodium carbonate (Na_2_CO_3_), tetrahydrofuran (THF), vanillin, sodium borohydride (NaBH_4_), methanol, and fluorescein disodium salt were purchased from Aladdin (Shanghai, China). Acetic acid, sodium hydroxide (NaOH), potassium hydroxide, potassium phosphate dibasic, potassium phosphate monobasic, aluminum chloride, and sodium bicarbonate (NaHCO_3_) were acquired from Sangon Company (Shanghai, China).

### 4.2. Germination Conditions

**Figure 5:**
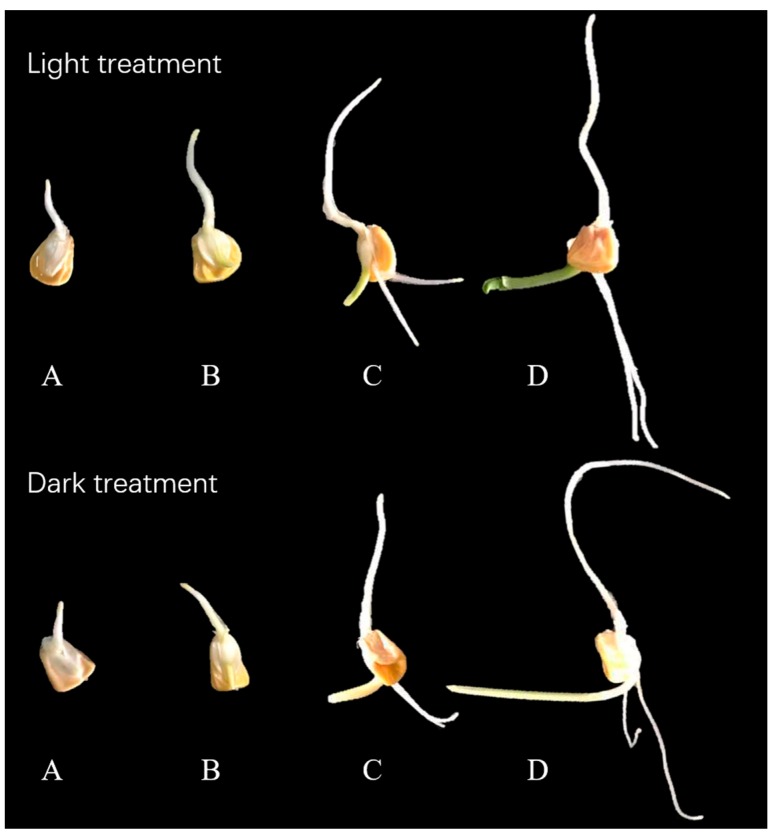
Sweet corn sprouts at different germination stages. (**A**) Radicle stage; (**B**) germinal stage; (**C**) fibril stage; and (**D**) euphylla stage.

Sweet corn seeds (common variety YT28) were provided by Jianguang Hu (Crops Research Institute of Guangdong Academy of Agricultural Sciences, Guangzhou, China), and soaked in 75% alcohol for 1 min to sterilize, and were then placed in distilled water for 2 h to stimulate germination. The seeds were then divided into light and dark groups, placed into culture dishes with wet degreasing cotton and filter paper, and kept in a growth cabinet exposed to light (24 h photoperiod, irradiance of 200 μmol·m^−2^·s^−1^) at 20 °C. In total, five replications were made for the light and the dark groups, respectively, each replication containing approximately 50 seeds. The dark group was enclosed with aluminized paper and stored at similar temperatures as the light group; however, the containers were kept in a darkened environment to avoid illumination. The germination percentages of both light- and dark-treated sprouts were 90%. Samples of the experimental sprouts for gene expression were selected from the five containers and pooled to achieve biological replications. For other measurements, the sprouts were selected from five containers, mixed, and divided into three replications. Collections of samples were determined by the germination period of the seed, which can be visually divided as radicle (the first appearance of the radicle), germinal (the first appearance of the germ), fibril (the first appearance of the fibril), and euphylla (the first appearance of the euphylla) stage. The morphological differences of sweet corn sprouts during germination are shown in Figure 5.

### 4.3. Moisture Content Measurement

The moisture content was measured using the modified oven-drying method [6]. Briefly, 2 g of the sweet corn sprouts or seedlings were oven dried at 105 °C to a constant weight. The measurements were performed as a percent of dry weight in triplicate and data were reported as mean ± SD (*n* = 3).

### 4.4. RNA Extractionand Gene Expression Quantitative Analysis

The procedure used was according to Wang et al. [29]. HiPure Plant RNA Mini Kits were purchased from Magen Company (Guangzhou, China). A PrimeScript™ RT Reagent Kit with gDNA Eraser and a SYBP^®^ Premix Ex Taq™ Kit were obtained from Takara Biotechnology (Dalian, China). The primers were designed by Primer3 software (Premier Biosoft, Palo Alto, CA, USA). The nucleotide sequences of primers were manufactured by Sangon Biotech Co., Ltd (Shanghai, China). The primers of *_Zm_PAL* (GenID: 542258) were: forward 5′GGATGGTGGAGGAGTACAGG′3 and reverse 5′CGGCATTGAGGAATCGGATG′3. The primers of *_Zm_4CL* (GenID: 100283944) were: forward 5′CGAGCAAGACTTGGACTTCG′3 and reverse 5′ATCAGGATGGTGTTGAGCGA′3. The primers of *_Zm_C4H* (GenID: 100282780) were: forward 5′TGTTCCGCATCATGTTCGAC′3 and reverse 5′GTTGTACTCGAAGCTCTGCG′3. The primers of internal gene *_Zm_ACT* (GenID: 100282267) were: forward 5′TGTGGCTTTGGGATCGTAGTC′3 and reverse 5′GAGCCACCGATCCAGACACT′3. *_Zm_ACT* is a widely used housekeeping gene [32] and is proved as a suitable reference gene for RT-PCR in maize, as reported previously [33]. Although the expressions of *ACT* were different during the germination period, as was reported before [34], the changes of expressions were identical with the target genes in our study. The total RNA was extracted from a frozen sample and continuously converted to DNase treatment and converted to cDNA. The concentration of mRNA and cDNA were quantitated before use. Real-time RT-PCR was accomplished using the LightCycler^®^ 480 Real-Time PCR System and the threshold cycle values were calculated using LightCycler^TM^ 480 software (F. Hoffmann-La Roche Ltd., Basel, Switzerland). The thermal cycle conditions were adjusted to be 3 min at 95 °C, followed by 40 cycles of amplification (5 s at 95 °C, 30 s at 56 °C, and 30 s at 72 °C). Each real-time PCR was followed by melt curve analysis to assess product specificity. The amplification results of each sample were analyzed as a cycle threshold (*C*t) value and the degree of transcription was calculated by the 2^−ΔΔ*C*t^ method. △△*C*_t_ = (*C*_t,Target_ − *C*_,Act_)_EG_ − (*C*_t,Target_ − *C*_,Act_)_CG_. The *C*_t_ values were kept stable during the germination period of sweet corn in our study. The sweet corn in the radicle stage was chosen as control group in both light and dark treatments. Results are reported as mean ± SE (*n* = 3).

### 4.5. Phytochemical Extraction

The phytochemicals of sweet corn sprouts were extracted by the following method, as reported previously [6]. Free phytochemicals were extracted with chilling with 80% (*v*/*v*) acetone. To extract bound phytochemicals, the residue from the free phytochemical extraction was digested with sodium hydroxide and acidified to pH 2 with hydrochloric acid. Extraction was following with ethyl acetate. All of the extracts were evaporated, dissolved with 70% alcohol, and maintained at −20 °C until determination.

### 4.6. Determination of Phenolics

Both the free and bound forms of phenolics were measured by the Folin-Ciocalteu colorimetric method with slight modification [6]. Briefly, extracts were diluted with Milli-Q water to make appropriate concentrations. Folin-Ciocalteu reagent was used to oxidize the extracts, and then the reaction was stopped with 7% Na_2_CO_3_. Gallic acid (GA) was used as the standard and the absorbance was measured at 760 nm. The concentrations were calculated based on the standard curve, and expressed as milligram Gallic acid equivalent per 100 grams of dry weight (mg GAE/100 g DW). Data were reported as mean ± SD for triplicate analysis.

### 4.7. Determination of Flavonoids

The free and bound flavonoid contents were determined using the sodium borohydride/chloranil protocol (SBC) method as reported previously [6]. Nitrogen gas was used to dry the solution. Tetrahydrofuran/ethanol (1:1, *v*/*v*) was added to reconstitute. The flavonoid content was expressed as milligrams catechin equivalent per 100 grams of dry weight of sample (mg CE/100 g DW). Data were reported as mean ± SD for three replicates.

### 4.8. Phenolic Compounds Analysis by HPLC

Gallic acid, chlorogenic acid, syringic acid, hydroxycinnamic acid, and ferulic acid were quantitatively determined using a Water series HPLC system equipped with a binary pump (model 1525) (Waters, Milford, MA, USA), a micro degasser, and an autosampler (model 2707) (Waters, Milford, MA, USA) as reported before [35]. Compounds were separated at 30 °C on a Water SunFire C18 column (4.6 mm × 250 mm, 5 μm) (Waters, Milford, MA, USA). (A) 0.1% trifluoroacetic acid (aqueous) and (B) methanol were used as mobile phases. The gradient elution was as follows: from 0 to 5 min, 10% B; from 5 to 20 min, 10% to 25% B; from 20 to 25 min, 25% to 35% B; from 25 to 40 min, 35% to 90% B; from 40 to 50 min, 90% to 10% B; from 50 to 60 min, 10% B. The flow rate was set at 1.0 mL/minute and the volume of injected sample was set as 20 μL. Each phenolic composition detected was expressed as milligrams per 100 grams of dry weight according to a standard curve of each substrate. Results were expressed as mean ± SD (*n* = 3).

### 4.9. Antioxidant Activity Assay

The oxygen radical absorbance capacity (ORAC) assay, as reported and modified previously, was used to determine antioxidant activity in the studied samples [36]. Trolox solution was used as the standard. Briefly, 200 μL fluoroscein working solution was added to each well of microplate which was then incubated at 37 °C for 20 min. The microplate was warmed to 37 °C, followed by the addition of 20 μL 2,2′-Azobis (2-methylpropionamidine) dihydrochloride (AAPH) working solution. The ORAC value was expressed as micro-mol Trolox equivalent per gram in dry weight (μmol TE/100 g DW). Data was reported as the mean ± SD for triplicate analysis.

### 4.10. Statistical Analysis

Statistical analyses were performed using Sigma Plot software 12.3 (Systat Software, Inc., Chicago, IL, USA). All measurements were performed in triplicate. Data were analyzed using Duncan’s multiple comparison post-test in light- and dark- treatments, respectively. A *p*-value < 0.05 was regarded as statistically significant. All statistical analyses of data were performed using SPSS for Windows version 18.0 (SPSS Inc., Chicago, IL, USA).

## 5. Conclusions

In summary, the total phenolic content, total flavonoid content, and antioxidant activity were increased during the germination of sweet corn seeds and reached the highest levels at the euphylla stage. Positive correlations were observed among TPC, TFC, and antioxidant activity during germination. The gene expression was associated with the biosynthetic pathways of these compounds. Compared with the dark group, exposure to light stimulated the generation of phenolics, which indicated a positive effect on the nutritional value of sweet corn. In conclusion, instead of consumption of fresh sweet corn kernels, germinated sweet corn sprouts under light to the euphylla stage suggest a healthier diet.

## Figures and Tables

**Table 1:** Changes in the main phenolic compounds during sweet corn germination.

Phenolic Compounds (mg/100 g DW)	Germination Stages	Light			Dark		
		Free Form	Bound Form	Total	Free Form	Bound Form	Total
Gallic acid	Radicle	83.33 ± 20.48a	26.20 ± 0.31a	109.5 ± 20.8a	81.84 ± 16.17a	28.96 ± 1.10a	110.8 ± 15.1a
	Germinal	115.36 ± 1.84a	33.09 ± 0.80a	135.4 ± 7.2b	75.14 ± 11.50a	36.16 ± 6.16a	111.3 ± 10.8a
	Fibril	93.10 ± 3.68ab	65.19 ± 11.89b	158.3 ± 14.8b	79.06 ± 15.37a	64.10 ± 7.05b	143.2 ± 20.3ab
	Euphylla	95.17 ± 20.93ab	74.40 ± 3.04b	169.6 ± 18.1b	93.83 ± 16.13a	63.88 ± 6.02b	157.7 ± 13.7b
Chlorogenic acid	Radicle	16.92 ± 0.18a	nd	16.92 ± 0.18a	16.39 ± 0.40a	nd	16.39 ± 0.40a
	Germinal	25.59 ± 0.78b	nd	25.59 ± 0.78b	22.00 ± 0.22b	nd	22.00 ± 0.22b
	Fibril	29.88 ± 1.74c	nd	29.88 ± 1.74c	29.33 ± 0.71c	nd	29.33 ± 0.71c
	Euphylla	40.72 ± 1.01d	nd	40.72 ± 1.01d	29.28 ± 0.42c	nd	29.28 ± 0.42c
Syringic acid	Radicle	13.19 ± 1.56a	nd	13.19 ± 1.56a	8.90 ± 0.15a	nd	8.90 ± 0.15a
	Germinal	21.98 ± 3.40b	nd	21.98 ± 3.40b	11.84 ± 1.19b	7.86 ± 0.03a	19.70 ± 1.18b
	Fibril	13.36 ± 1.06a	9.85 ± 0.88a	23.21 ± 1.86b	12.51 ± 1.18b	9.56 ± 0.15b	22.07 ± 1.05c
	Euphylla	15.67 ± 0.15a	12.69 ± 0.17b	28.36 ± 0.08c	11.40 ± 0.57b	10.74 ± 0.22c	22.14 ± 0.76c
Hydroxycinnamic acid	Radicle	13.69 ± 0.25a	60.73 ± 2.86a	74.42 ± 2.87a	13.12 ± 0.31a	51.37 ± 0.68a	64.49 ± 0.88a
	Germinal	17.15 ± 0.27b	73.23 ± 7.19b	90.38 ± 10.18b	15.51 ± 0.18b	58.84 ± 1.31b	74.35 ± 1.49b
	Fibril	18.26 ± 1.73b	74.15 ± 4.43b	92.41 ± 6.15b	19.61 ± 0.15c	72.13 ± 1.79c	91.74 ± 1.92c
	Euphylla	24.86 ± 0.36c	109.3 ± 2.3c	134.1 ± 2.6c	21.31 ± 0.22d	101.3 ± 2.9d	122.6 ± 2.7d
Ferulic acid	Radicle	14.48 ± 0.37a	290.5 ± 0.8a	305.0 ± 0.5a	13.99 ± 0.62a	256.7 ± 9.5ab	270.7 ± 9.9a
	Germinal	18.17 ± 0.05b	342.0 ± 37.0b	360.2 ± 37.0b	15.96 ± 0.19b	246.1 ± 7.8a	262.1 ± 7.9a
	Fibril	19.78 ± 1.83c	312.1 ± 9.9ab	331.9 ± 11.8ab	20.04 ± 0.24c	284.5 ± 5.6c	304.5 ± 5.8b
	Euphylla	27.30 ± 0.36d	318.4 ± 6.0ab	345.7 ± 5.7b	22.12 ± 0.29d	269.0 ± 11.5bc	291.1 ± 11.2b

Values with no letters in common in each column are significantly different (*p* < 0.05). “nd” means “not detected”.

## References

[1] Chen G., Wang H., Zhang X., Yang S.T. (2014). Nutraceuticals and functional foods in the management of hyperlipidemia. Crit. Rev. Food Sci. Nutr..

[2] Liu R.H. (2004). Potential synergy of phytochemicals in cancer prevention: Mechanism of action. J. Nutr..

[3] Ramful D., Tarnus E., Aruoma O.I., Bourdon E., Bahorun T. (2011). Polyphenol composition, vitamin C content and antioxidant capacity of *Mauritian citrus* fruit pulps. Food Res. Int..

[4] Pientaweeratch S., Panapisal V., Tansirikongkol A. (2016). Antioxidant, anti-collagenase and anti-elastase activities of *Phyllanthus emblica*, *Manilkara zapota* and silymarin: An in vitro comparative study for anti-aging applications. Pharm. Biol..

[5] Spencer J.P.E., Vafeiadou K., Williams R.J., Vauzour D. (2012). Neuroinflammation: Modulation by flavonoids and mechanisms of action. Mol. Asp. Med..

[6] Guo X., Li T., Tang K., Liu R.H. (2012). Effect of germination on phytochemical profiles and antioxidant activity of mung bean sprouts (*Vigna radiata*). J. Agric. Food Chem..

[7] Song J.F., Liu C.Q., Li D.J., Meng L.L. (2013). Effect of cooking methods on total phenolic and carotenoid amounts and DPPH radical scavenging activity of fresh and frozen sweet corn (*Zea mays*) kernels. Czech J. Food Sci..

[8] Sylvia M. Burwell, Thomas J. Vilsack, U.S. Department of Health and Human Services, U.S. Department of Agriculture Dietary Guidelines for Americans 2015–2020, Eighth Edition. www.dietaryguidelines.gov.

[9] Fahey J.W., Zhang Y.S., Talalay P. (1997). Broccoli sprouts: An exceptionally rich source of inducers of enzymes that protect against chemical carcinogens. Proc. Natl. Acad. Sci. USA.

[10] Gan R.Y., Lui W.Y., Wu K., Corke H. (2016). Thermal treatments affect the polyphenol profile and increase antioxidant capacity in five varieties of edible bean milks. Int. J. Food Sci. Technol..

[11] Świeca M., Dziki D. (2015). Improvement in sprouted wheat flour functionality: Effect of time, temperature and elicitation. Int. J. Food Sci. Technol..

[12] Haileslassie H.A., Henry C.J., Tyler R.T. (2016). Impact of household food processing strategies on antinutrient (phytate, tannin and polyphenol) contents of chickpeas (*Cicer arietinum* L.) and beans (*Phaseolus vulgaris* L.): A review. Int. J. Food Sci. Technol..

[13] Ti H., Zhang R., Zhang M., Li Q., Wei Z., Zhang Y., Tang X., Deng Y., Liu L., Ma Y. (2014). Dynamic changes in the free and bound phenolic compounds and antioxidant activity of brown rice at different germination stages. Food Chem..

[14] Ren S.-C., Sun J.-T. (2014). Changes in phenolic content, phenylalanine ammonia-lyase (PAL) activity, and antioxidant capacity of two buckwheat sprouts in relation to germination. J. Funct. Foods.

[15] Wang L., Wang H., Lai Q., Li T., Fu X., Guo X., Liu R.H. (2015). The dynamic changes of ascorbic acid, tocopherols and antioxidant activity during germination of soya bean (*Glycine max*). Int. J. Food Sci. Technol..

[16] Majid I., Dhatt A.S., Sharma S., Nayik G.A., Nanda V. (2016). Effect of sprouting on physicochemical, antioxidant and flavonoid profile of onion varieties. Int. J. Food Sci. Technol..

[17] Duenas M., Martinez-Villaluenga C., Limon R.I., Penas E., Frias J. (2015). Effect of germination and elicitation on phenolic composition and bioactivity of kidney beans. Food Res. Int..

[18] Valcarcel J., Reilly K., Gaffney M., O′Brien N.M. (2016). Levels of potential bioactive compounds including carotenoids, vitamin C and phenolic compounds, and expression of their cognate biosynthetic genes vary significantly in different varieties of potato (*Solanum tuberosum* L.) grown under uniform cultural conditions. J. Sci. Food Agric..

[19] Han C., Li J., Jin P., Li X., Wang L., Zheng Y. (2017). The effect of temperature on phenolic content in wounded carrots. Food Chem..

[20] Wang L., Li X., Niu M., Wang R., Chen Z. (2013). Effect of additives on flavonoids, d-chiro-Inositol and trypsin inhibitor during the germination of tartary buckwheat seeds. J. Cereal Sci..

[21] Yu D., Bu F., Hou J., Kang Y., Yu Z. (2016). A morel improved growth and suppressed Fusarium infection in sweet corn. World J. Microbiol. Biotechnol..

[22] Randhir R., Shetty K. (2005). Developmental stimulation of total phenolics and related antioxidant activity in light- and dark-germinated corn by natural elicitors. Process Biochem..

[23] Chen Z., Yu L., Wang X., Gu Z., Beta T. (2016). Changes of phenolic profiles and antioxidant activity in canaryseed (*Phalaris canariensis* L.) during germination. Food Chem..

[24] Winkelshirley B. (2001). Flavonoid biosynthesis. A Colorful model for genetics, biochemistry, cell biology, and biotechnology. Plant Physiol..

[25] Dewanto V., Wu X.Z., Liu R.H. (2002). Processed sweet corn has higher antioxidant activity. J. Agric. Food Chem..

[26] Sritongtae B., Sangsukiam T., Morgan M.R.A., Duangmal K. (2017). Effect of acid pretreatment and the germination period on the composition and antioxidant activity of rice bean (*Vigna umbellata*). Food Chem..

[27] Cheynier V., Comte G., Davies K.M., Lattanzio V., Martens S. (2013). Plant phenolics: Recent advances on their biosynthesis, genetics, and ecophysiology. Plant Physiol. Biochem..

[28] Peters N.K., Verma D.P. (1990). Phenolic compounds as regulators of gene expression in plant-microbe relations. Mol. Plant Microbe Interact..

[29] Wang H., Wang J., Guo X., Brennan C.S., Li T., Fu X., Chen G., Liu R.H. (2016). Effect of germination on lignan biosynthesis, and antioxidant and antiproliferative activities in flaxseed (*Linum usitatissimum* L.). Food Chem..

[30] Liu H., Chen Y., Hu T., Zhang S., Zhang Y., Zhao T., Yu H., Kang Y. (2016). The influence of light-emitting diodes on the phenolic compounds and antioxidant activities in pea sprouts. J. Funct. Foods.

[31] Seo J.-M., Arasu M.V., Kim Y.-B., Park S.U., Kim S.-J. (2015). Phenylalanine and LED lights enhance phenolic compound production in Tartary buckwheat sprouts. Food Chem..

[32] Lin F., Jiang L., Liu Y., Lv Y., Dai H., Zhao H. (2014). Genome-wide identification of housekeeping genes in maize. Plant Mol. Biol..

[33] Galli V., Messias R.d.S., dos Anjos e Silva S.D., Rombaldi C.V. (2013). Selection of reliable reference genes for quantitative real-time polymerase chain reaction studies in maize grains. Plant Cell Rep..

[34] Díaz-Camino C., Conde R., Ovsenek N., Villanueva M.A. (2005). Actin expression is induced and three isoforms are differentially expressed during germination in *Zea mays*. J. Exp. Bot..

[35] Zhang L.Z., Liu R.H. (2015). Phenolic and carotenoid profiles and antiproliferative activity of foxtail millet. Food Chem..

[36] Prior R.L., Sintara M., Chang T. (2016). Multi-radical (ORACMR5) antioxidant capacity of selected berries and effects of food processing. J. Berry Res..

